# Dataset of the *HOX1* gene sequences of the wheat polyploids and their diploid relatives

**DOI:** 10.1016/j.dib.2017.11.010

**Published:** 2017-11-07

**Authors:** Andrey B. Shcherban, Elena A. Salina

**Affiliations:** The Federal Research Center “The Institute of Cytology and Genetics SB RAS”, Russian Federation

**Keywords:** Wheat, Polyploid, *HOX-1* gene, Homeodomain, Transcription factor, Promoter, Triticum, Aegilops

## Abstract

The *TaHOX-1* gene of common wheat *Triticum aestivum* L. (BAD-genome) encodes transcription factor (HD-Zip I) which is characterized by the presence of a DNA-binding homeodomain (HD) with an adjacent Leucine zipper (LZ) motif. This gene can play a role in adapting plant to a variety of abiotic stresses, such as drought, cold, salinity etc., which strongly affect wheat production. However, it's both functional role in stress resistance and divergence during wheat evolution has not yet been elucidated. This data in brief article is associated with the research paper “Structural and functional divergence of homoeologous copies of the *TaHOX-1* gene in polyploid wheats and their diploid ancestors”. The data set represents a recent survey of the primary *HOX-1* gene sequences isolated from the first wheat allotetraploids (BA-genome) and their corresponding *Triticum* and *Aegilops* diploid relatives. Specifically, we provide detailed information about the *HOX-1* nucleotide sequences of the promoter region and both nucleotide and amino acid sequences of the gene. The sequencing data used here is available at DDBJ/EMBL/GenBank under the accession numbers MG000630-MG000698.

**Specifications Table**

**Table:** 

Subject area	*Biology*
More specific subject area	*Molecular genomics of plants*
Type of data	*Genomic DNA sequencing data*
How data was acquired	*Sequencing was performed in an ABI PRISM 310 Genetic Analyzer (Perkin 443 Elmer Cetus)*
Data format	*Raw sequences (fastq), analyzed sequences (figures)*
Experimental factors	*Non-treated seedlings*
Experimental features	*Total genomic DNA was extracted from one week old etiolated seedlings grown at room temperature from seeds placed in wet filter paper in Petri dishes. Using genomic DNA as a template, PCR amplification of the HOX-1 gene fragments was performed followed by their sequencing and computer analysis.*
Data source location	*N.A.*
Data accessibility	*The HOX-1 sequences of the allotetraploid wheat species and their diploid relatives were deposited in the NCBI database under accession No.*MG000630-MG000698

**Value of the data**

• Analysis of gene networks which control plant growth depending on environmental conditions is prerequisite for improvement of production of such economically valuable plants as wheat under fluctuations in water status, light conditions, nutrient status, temperature etc.
• The homeodomain-leucine zipper HD-Zip I transcription factor network regulate the plant growth in response to environmental stimuli.
• Structural characterization of the genes encoding HD-Zip I (*Hox-1*) in polyploid wheats and their diploid relatives is important to unravel how the molecular mechanisms underlying sensitivity of plants to environmental factors evolved during formation of allopolyploid species from their diploid predecessors.

## 1 Data

**Fig. 1:**
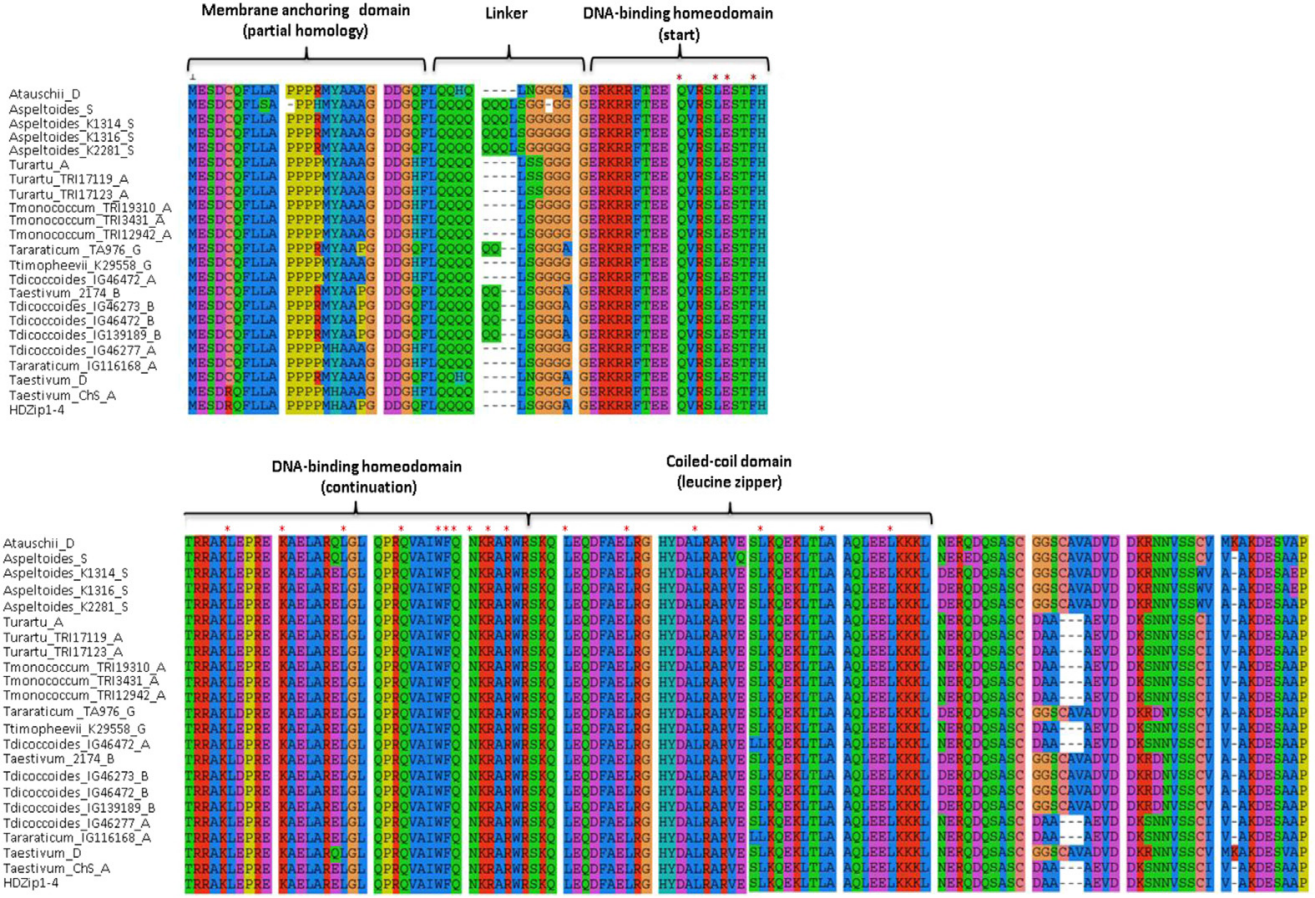
A multiple sequence alignment of the studied protein *HOX-1* sequences. The basic functional domains are shown above the alignment. Red asterisks indicate the most conservative residues within domains. The most of sequences were isolated in this work, except those downloaded from NCBI: Atauschii_D (XP_020152926), Turartu_A (EMS54941), Taestivum_2174_B (AGC26413), HDZip1-4 (AMB42697) and URGI database (https://wheat-urgi.versailles.inra.fr/Seq-Repository/): Aspeltoides_S (TGAC_WGS_speltoides_v1_ contig_201042), Taestivum_D (TGACv1_ scaffold_526953), Taestivum_ChS_A (TGACv1_ scaffold_439821).

**Fig. 2:**
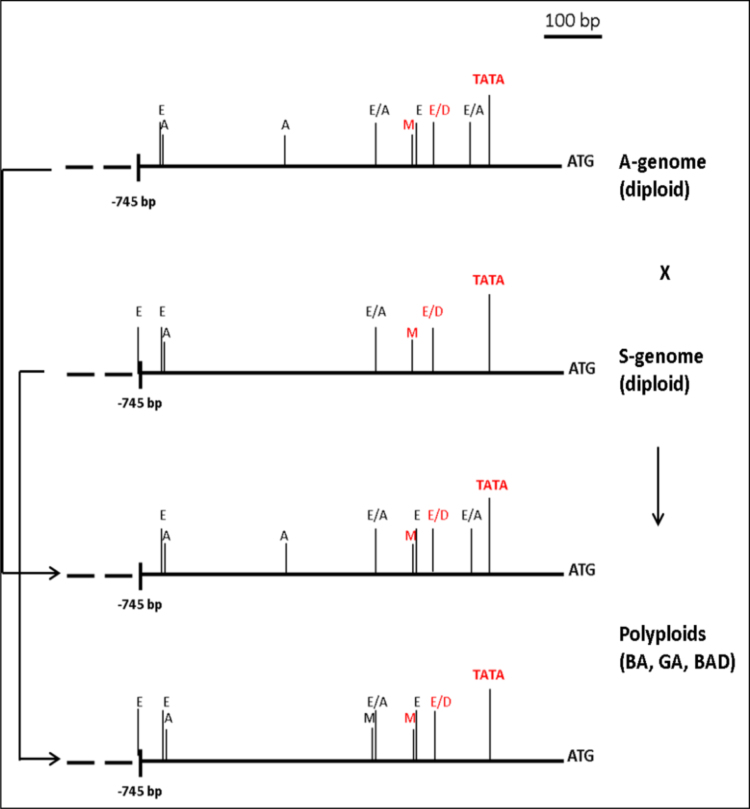
Schematic presentation of 0.7 kb promoter region of *HOX-1* in diploid species with A- and S- genomes and corresponding genomes of polyploid wheats (BA, GA and BAD genomes). Dotted line denotes highly variable region. ATG, the start codon. TATA, the putative TATA-box. The putative cis- regulatory elements, associated with response to drought (Dr) and/or abscisic acid (ABA) are shown: E- EBOXBNNAPA (ABA); D- DPBFCOREDCDC3 (ABA), A- ACGTATERD1 (Dr); M- MYB (ABA, Dr). The most conservative elements (also present in *OsHOX24* gene of rice) are in red.

**Fig. 3:**
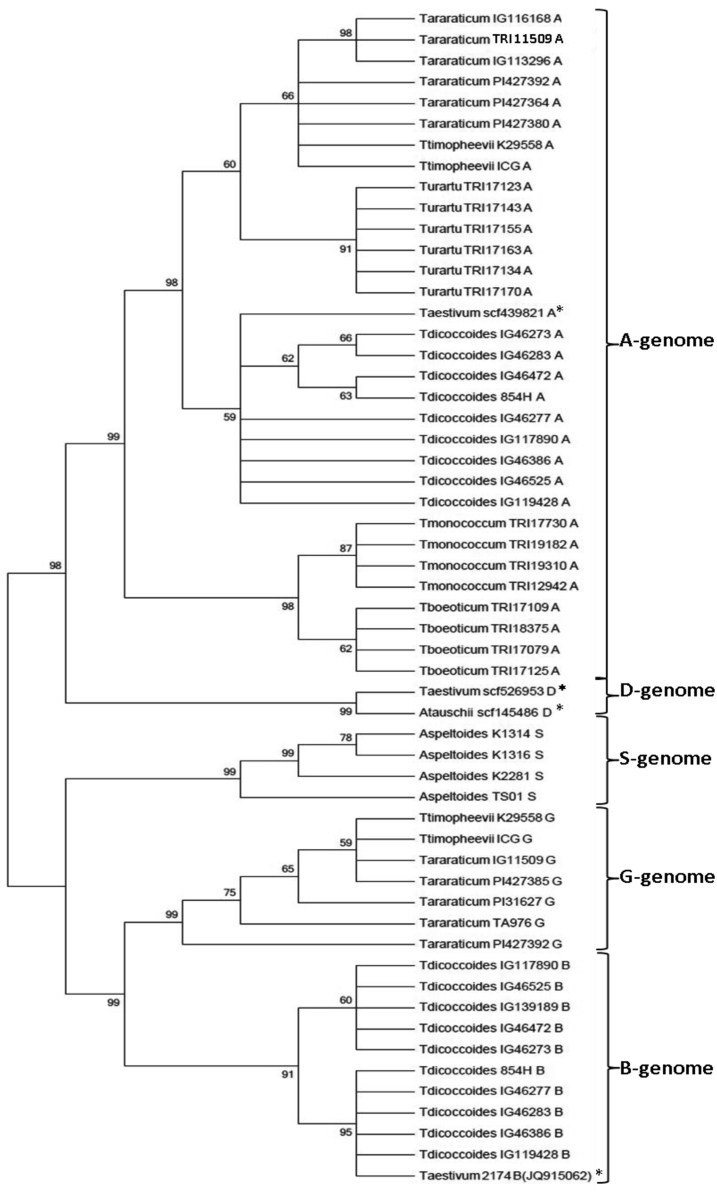
The neighbor-joining tree based on the alignment of the nucleotide *HOX-1* promoter sequences. The numbers above or below forks indicate bootstrap values. Asterisks mark the sequences downloaded from databases.

The data include a list of species/accessions used in this study (Table 1), a multiple sequence alignment of the studied protein *HOX-1* sequences with indication of basic structural domains (Fig. 1), schematic representation of 0.7 kb promoter region of *HOX-1* in diploid species with A- and S- genomes and corresponding genomes of polyploid wheats (Fig. 2), the neighbor-joining tree based on the alignment of the nucleotide *HOX-1* promoter sequences (Fig. 3). The nucleotide and amino acid *HOX-1* sequences from different accessions are available in fasta- format as Supplementary material 1.

**Table 1:** Plant material used in the analysis.

Species/Accession no.	Genome	Origin	Source^a^
***Triticum monococcum*****L.**			
TRI 3431	AA	Austria	IPK
TRI 17730	AA	Turkey	IPK
TRI 19182	AA	Morocco	IPK
TRI 19310	AA	Albania	IPK
TRI 12942	AA	France	IPK
***Triticum boeoticum*****Boiss.**			
TRI 17109	AA	Iraq	IPK
TRI 18375	AA	Iraq	IPK
TRI 17079	AA	Turkey	IPK
TRI 17125	AA	Turkey	IPK

***Triticum urartu*****Thum ex Gandil.**			
TRI 17123	AA	Turkey	IPK
TRI 17143	AA	Lebanon	IPK
TRI 17155	AA	Lebanon	IPK
TRI 17163	AA	Lebanon	IPK
TRI 17134	AA	Turkey	IPK
TRI 17170	AA	Turkey	IPK
TRI 17119	AA	Turkey	IPK
***Aegilops speltoides*****Tausch.**			
K-1314	SS	Israel	VIR
K-1316	SS	Israel	VIR
K-2281	SS	**Unknown**	VIR
TS01	SS	Israel	WIC
***Triticum dicoccoides*****Thell.**			
854H	BBAA	Israel	WIC
IG 46273	BBAA	Israel	ICARDA
IG 46283	BBAA	Israel	ICARDA
IG 46472	BBAA	Syria	ICARDA
IG 46277	BBAA	Israel	ICARDA
IG 117890	BBAA	Syria	ICARDA
IG 46386	BBAA	Jordan	ICARDA
IG 46525	BBAA	Syria	ICARDA
IG 119428	BBAA	Syria	ICARDA
IG 139189	BBAA	Jordan	ICARDA
***T. araraticum*****Jakubz.**			
IG 116168	GGAA	Turkey	ICARDA
TRI 11509	GGAA	Iran	IPK
IG 113296	GGAA	Iran	ICARDA
PI 427392	GGAA	Iraq	USDA-ARS
PI 427364	GGAA	Iraq	USDA-ARS
PI 427380	GGAA	Iraq	USDA-ARS
PI 427385	GGAA	Iraq	USDA-ARS
K-31627	GGAA	Azerbaijan	VIR
TA 976	GGAA	Turkey	WGGR,KSU
***T. timopheevii*****(Zhuk) Zhuk. (ssp.*****T. araraticum*****)**			
K-29558	GGAA	Georgia	VIR
ICG	GGAA	Unknown, provided by E.B.Budashkina	Institute of Cytology and Genetics SB RAS

a USDA-ARS- United States Department of Agriculture, Agricultural Research Service; WGGR, KSU- The Wheat GermPlasm Collection of Kansas State University, USA; VIR- N. I. Vavilov All-Union Research Institute of Plant Industry, St Petersburg, Russia; IPK- The Leibniz Institute of Plant Genetics and Crop Plant Research, Gatersleben, Germany; ICARDA- International Center for Agricultural Research in the Dry Areas; WIC- Weizmann Institute of Science Collection, Rehovot, Israel.

## 2 Experimental design, materials and methods

### 2.1 Plant Material and DNA extraction

As a material we used a set of accessions (3–10 accessions per species) representing tetraploid (2n = 28) wheat species *T. dicoccoides* (BA), *T. araraticum*/ *timopheevii* (*GA*), as well as diploid (2n = 14) species: 1) *T. monococcum*/ *boeoticum*, *T. urartu*, a putative donors of А- genome, and 2) *Ae. speltoides* (SS), a putative donor of B/G- genomes to wheat polyploids (Table 1). DNA was extracted from 7-day-old seedlings following [1]. Leaves from 3–5 seeds per accession were homogenised using a FastPrep-24 instrument (MP Biomedicals, USA).

### 2.2 PCR

In order to amplify the promoter and gene sequences of *HOX-1*, specific primers were constructed based on the homoeologous (related to different subgenomes) copies of this gene *TaHOX-A1*, *TaHOX-B1*, downloaded from databases (see legend to Fig. 1). Specific forward primers for the *HOX-1* promoter region related to A and B(G)- genomes were HOX1AF (5′-AGTCCAACTGTCCAACTGATGG-3′), HOX1BF (5′-GAACTTGACATGAGCAGCGG-3′), respectively. In the case of *Ae. speltoides* the forward primer was HOX1SF (5′-GCTTCGATCGGCGCCACGTT-3′). These genome-specific primers were combined with the same reverse primer HOX1R (5′-CAGTCGCTCTCCATTTCGGA-3′), overlapping the start ATG-codon. Specific forward primers for amplification of the *HOX-1* coding region related to A and B(G)/S- genomes were HOXCOD1AF (5′-CGCCACAGATGCACGCCTGG-3′), HOXCOD1BF (5′-ACCACGTTCCAAACGCCACC-3′), respectively. These genome-specific primers were combined with the same reverse primer HOXCOD1R (5′-TCATGCCACTGCGTTCCACTCC-3′). PCR was performed using a DNA Thermal Cycler 480 (Perkin Elmer Cetus, USA). Reaction mixtures were in a volume of 20 µl containing 50–100 ng of genomic template DNA, 1 ng of each of primer, 0.25 mM of each dNTP, 1x reaction buffer (67 mM TrisHCl, pH 8.8; 2 mM MgCl_2_; 18 mM (NH_4_)_2_SO_4_; 0.01% Tween 20) and 1 unit *Taq* polymerase. After initial denaturation at 94 °C for 2 min, 35 cycles were run at 94 °C for 1 min, 55–60 °C (depending on the primer pair used) for 1 min, and 72 °C for 1 min, followed by a final extension at 72 °C for 5 min. PCR products were separated on 1% agarose gel, stained with ethidium bromide and visualized under UV light.

### 2.3 Isolation and sequencing of PCR products

The PCR products were excised from the gel and purified using a QIAquick PCR purification kit (QIAGEN, Germany), then directly sequenced in both directions using an ABI PRISM Dye Terminator Cycle Sequencing ready reaction kit (Perkin Elmer Cetus, USA). Sequencing was conducted using resources of SB RAS Genomics Core Facilities (Novosibirsk, Russia, http://sequest.niboch.nsc.ru).

### 2.4 Sequence analysis

The nucleotide sequences were aligned using the ClustalW program with the MEGA4 software package [2], [3]. Based on the known HDZip1 protein (AMB42697), the coding *HOX-1* sequences were translated with subsequent alignment of a selective set of structurally different amino acid sequences for each species (Fig. 3). The putative cis- regulatory, stress responsive elements in the gene promoter were searched using database PlantPAN 2.0 (http://plantpan2.itps.ncku.edu.tw). Fig. 2 represents the most conservative elements implicated in response to drought and/or abscisic acid (ABA) which triggers ABA signaling pathway associated with abiotic stress.

Based on the alignment of *HOX-1* promoter sequences, a phylogenetic tree was constructed by the neighbor-joining method, using 500 bootstrap replicates and pairwise deletion of gaps (Fig. 3).

The *HOX-1* promoter and coding sequences (including exons 1, 2 and intervening intron) were deposited to GenBank (https://www.ncbi.nlm.nih.gov/) under Ac. nos. MG000630-81 and MG000682-98, respectively.

## Appendix A Supplementary material

Supplementary material **Supplementary_file1** Nucleotide sequences of the *HOX1* promoter region ~ 740 bp from ATG-codon.

Supplementary material **Supplementary_file2** Nucleotide sequences of the *HOX1* coding region including 2 exons and intervening intron (~ 120 bp).

Supplementary material **Supplementary_file3** Amino acid sequences of *HOX1*.

## Footnotes

Appendix A Supplementary data associated with this article can be found in the online version at https://doi.org/10.1016/j.dib.2017.11.010.
